# Supplementary material for: Metabolic Dysfunction-Associated Steatotic Liver Disease in a Dish: Human Precision-Cut Liver Slices as a Platform for Drug Screening and Interventions
Source: Nutrients. 2024 Feb 23;16(5):626. doi: 10.3390/nu16050626 (PMC10934612; doi:10.3390/nu16050626)
Supplement: Supplementary file 1 [file nutrients-16-00626-s001.zip › Figure S1.pdf]

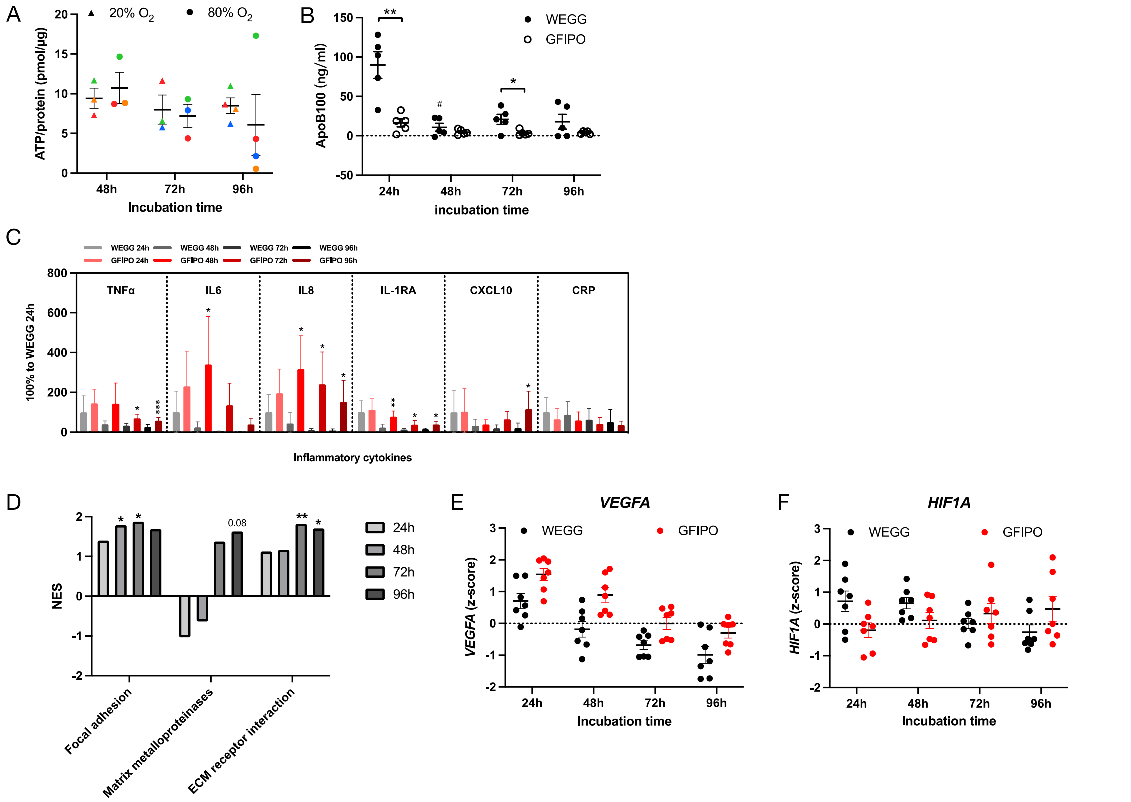

Figure S1: Altered ATP, ApoB100, pre-selected biological pathways, and gene expression in PCLSs. A ATP/protein content in human PCLSs after up to 96 h incubation in WEGG medium, under either 20 or 80% O_2. Different colors represent different livers. **B** ApoB100 secretion by PCLSs after incubation with WEGG and GFIPO. (*) denotes statistical differences between GFIPO and WEGG at each time point, while (#) denotes statistical differences in GFIPO or WEGG compared to their corresponding 24 h; *(#)*p* < 0.05, **(##)*p* < 0.01. **C** Bar chart showing secretion of inflammatory cytokines by PCLSs after up to 96 h of incubation (100% to WEGG 24 h). Data are presented as mean ± SD, (*) denotes statistical differences between GFIPO and WEGG at each time point; *p < 0.05, **p < 0.01, ***p < 0.001. **D** Altered “Focal adhesion”, “Matrix metalloproteinases”, and “ECM receptor interaction” pathways by GFIPO compared to WEGG at each time point (*/** indicates significantly changed compared to the corresponding WEGG, which was calculated by GSEA, **p.adj* < 0.05, ***p.adj* < 0.01). **E-F** Gene expression of *VEGFA* (E) and *HIF1A* (F) displayed in z-score by NGS.
