# Supplementary material for: Metabolic Dysfunction-Associated Steatotic Liver Disease in a Dish: Human Precision-Cut Liver Slices as a Platform for Drug Screening and Interventions
Source: Nutrients. 2024 Feb 23;16(5):626. doi: 10.3390/nu16050626 (PMC10934612; doi:10.3390/nu16050626)
Supplement: Supplementary file 1 [file nutrients-16-00626-s001.zip › Figure S2.pdf]

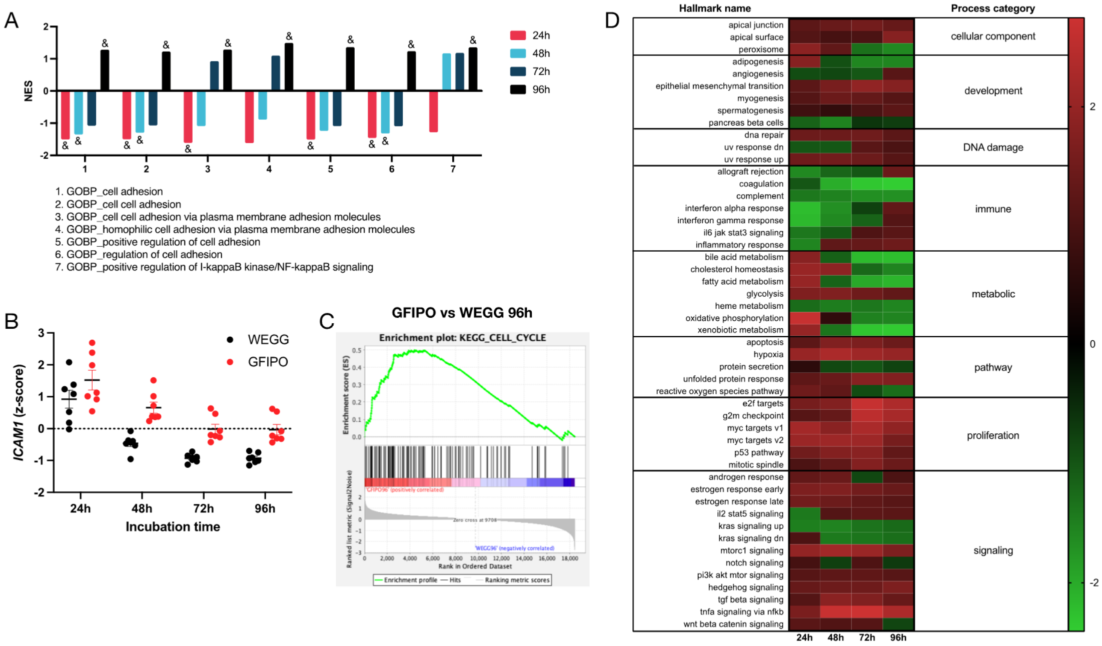

Figure S2: Altered biological processes or pathways and ICAM1 gene expression in PCLSs. **A** Altered GO biological processes by GFIPO compared to WEGG at each time point (& indicates significantly changed compared to the corresponding WEGG, which was calculated by GSEA). **B** Gene expression of *ICAM1* displayed in z-score by NGS. **C** GSEA plot showing the up-regulated KEGG pathway cell cycle at 96 h by GFIPO compared to WEGG. **D** Heatmap of all altered hallmark pathways by GFIPO compared to WEGG (red: up-regulated; green: down-regulated).
