# Supplementary material for: Metabolic Dysfunction-Associated Steatotic Liver Disease in a Dish: Human Precision-Cut Liver Slices as a Platform for Drug Screening and Interventions
Source: Nutrients. 2024 Feb 23;16(5):626. doi: 10.3390/nu16050626 (PMC10934612; doi:10.3390/nu16050626)
Supplement: Supplementary file 1 [file nutrients-16-00626-s001.zip › Table S1.pdf]

Table S1: List of SYBR Green primers used for RT-qPCR

Primers	Forward sequence (5'-3')	Reverse sequence (5'-3')
TNF	GAGGCCAAGCCCTGGTATG	CGGGCCGATTGATCTCAGC
IL6	ACTCACCTCTTCAGAACGAATTG	CCATCTTTGGAAGGTTCAGGTTG
IL1β	TACCTGTCCTGCGTGTTGAA	TCTTTGGGTAATTTTTGGGATCT
COL1A1	CAATCACCTGCGTACAGAACGCC	CGGCAGGGCTCGGGTTTC
ACTA2	AAAAGACAGCTACGTGGGTGA	GCCATGTTCTATCGGGTACTTC
MKI67	CTCAGCACCTGCTTGTTTGG	TTGCCTCCTGCTCATGGATT
